# ICAM-1 regulates macrophage polarization by suppressing MCP-1 expression via miR-124 upregulation

**DOI:** 10.18632/oncotarget.22948

**Published:** 2017-12-05

**Authors:** Wei Gu, Lun Yao, Lexing Li, Jianping Zhang, Aaron T. Place, Richard D. Minshall, Guoquan Liu

**Affiliations:** ^1^ Department of Basic Veterinary Medicine, College of Animal Science and Veterinary Medicine, Huazhong Agricultural University, Wuhan, Hubei Province 430070, P.R. China; ^2^ Department of Pharmacology, College of Medicine, University of Illinois at Chicago, Chicago, IL 60612, USA; ^3^ Department of Anesthesiology, College of Medicine, University of Illinois at Chicago, Chicago, IL 60612, USA

**Keywords:** ICAM-1, miR-124, transcript regulation, macrophage polarization, lung injury

## Abstract

Intercellular adhesion molecule-1 is the adhesion molecule mediating leukocyte firm adhesion to endothelial cells, plays a critical role in subsequent leukocyte transmigration. ICAM-1 is also expressed in other cells including macrophages; however, the role of this adhesion molecule in mediating macrophage functions remains enigmatic. We report that ICAM-1 regulates macrophage polarization by positively modulating miR-124 expression. We found higher expression levels of monocyte chemotactic protein-1 in lungs of mice lacking ICAM-1. Consistent with this result, siRNA mediated depletion of ICAM-1 in macrophage resulted in increased expression levels of MCP-1. Moreover, ICAM-1 controlled miR-124 expression and downregulated MCP-1 mRNA and protein expression by binding of miR-124 to MCP-1 3’ untranslated region. ICAM-1 also induced the transcription factor Sp1 expression, which is important for miR-124 expressing in macrophages. Furthermore, ICAM-1 depletion led to M1 macrophage polarization, in contrast, miR-124 mimics promoted M2 macrophage polarization. Exogenous administration of miR-124 mimics into the lungs prevented lipopolysaccharide-induced myeloperoxidase activity *in vivo*, suggesting that miR-124 is important for dampening acute lung injury. These results collectively show that adhesion molecule ICAM-1 downregulates MCP-1 expression by controlling Sp1 mediated miR-124 levels, which in turn regulate M2 macrophage polarization. Targeting ICAM-1 and downstream miR-124 may present a new therapeutic strategy for acute lung injury.

## INTRODUCTION

Upon infection and inflammation, cytokines and chemokines secreted by affected tissue guide leukocytes recruitment [1, 2]. The recruited leukocytes follow several well-defined and orchestrated steps including rolling, firm adhesion, and diapedesis to migrate across the endothelial barrier into the affected tissue. Among specific molecular interactions between endothelial cells and leukocytes responsible for these actions, intercellular adhesion molecule (ICAM-1) is the primary endothelial cell adhesion molecule mediating the firm adhesion of leukocytes and subsequent leukocyte transmigration [3, 4].

It is well known that pro-inflammatory cytokines increase ICAM-1 expression in endothelial cells in an NFκB-dependent manner, and ICAM-1 activation by integrin molecules on the leukocyte surface leads to outside-in signaling in endothelial cells [5, 6]. In addition, our previous study suggested that ICAM-1 is involved in the regulation of monocyte chemotactic protein-1 (MCP-1) expression in endothelial cells [7]. Nevertheless, ICAM-1 is also expressed in other cells including epithelial cells and macrophages [8, 9]. In epithelial cells, in addition to mediating the transmigration of leukocyte across-epithelial cells, ICAM-1 also acts as a receptor for human rhinovirus [10]. However, the role of ICAM-1 in macrophage remains relatively less clear. Recently it is suggested that ICAM-1 may regulate macrophage polarization and suppress tumor metastasis [11].

MicroRNAs (miRNAs) are 18∼23bp small RNA molecules, playing vital roles in the regulation of gene expression, cell differentiation and cell proliferation developmental processes [12]. MiR-124 was first discovered in mouse brain tissue [13]. In neurons enriched miR-124 inhibits the expression of small C-terminal domain phosphatase 1 (SCP-1), a key regulator to the process of induction of neuronal differentiation [14]. In cervical cancer cells or hepatocellular carcinoma cells, overexpression of miR-124 suppressed the occurrence of tumors [15, 16]. It is known that miR-124 targets transcription factor STAT3, regulating of endometrial carcinoma cell cycle G1 phase and then inhibiting tumor differentiation [17]. Recent studies show that in the pathological pulmonary hypertension, miR-124 also controls pulmonary vascular fibroblast proliferation, migration and inflammation [18] by inhibiting the expression of MCP-1 and polypyrimidine tract-bind protein 1(PTBP-1). Moreover, high level of miR-124 expression induces M2 macrophage polarization in the patients with bronchial asthma [19]. Clearly, the roles of miR-124 in macrophage and inflammation need further study.

Our previous study shows that ICAM-1 expression negatively correlates with MCP-1expression *in virus* infected swine lungs [20]. In this report, we observed the overexpression of MCP-1 in ICAM-1 knockout mouse lungs and found that miR-124 mediates ICAM-1 regulated MCP-1 expression in macrophages, thereby modulates macrophage polarization. Our studies thus identify a previously unrecognized pathway of ICAM-1/miR-124/MCP-1 in regulate macrophage polarization.

## RESULTS

### MCP-1 expression is elevated in the lungs of ICAM-1 knockout mice

**Figure 1:**
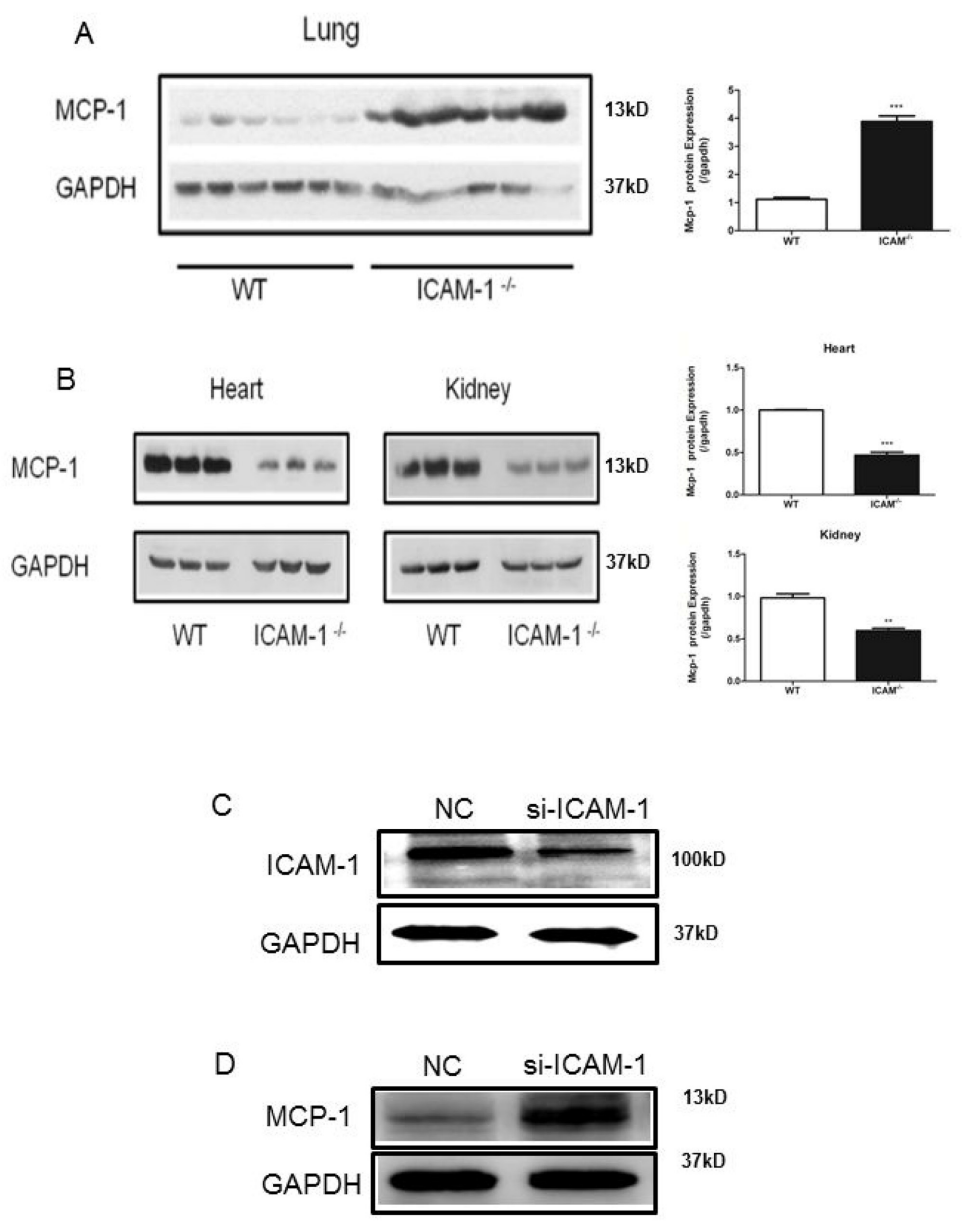
ICAM-1 deficiency or downregulation promotes MCP-1 expression in mouse lungs and macrophages **(A)** Comparison of MCP-1 expression in WT or ICAM-1^-/-^ mouse lungs by Western blot analysis. MCP-1 is elevated in ICAM-1^-/-^ mouse lungs. **(B)** Comparison of MCP-1 expression in WT or ICAM-1^-/-^ mouse heart and kidney. MCP-1 expression is relative decreased. **(C)** In RAW264.7 cells transfected with siRNA-ICAM-1, knockdown of ICAM-1 was measured with Western blot analysis. **(D)** In RAW264.7 transfected with siRNA-ICAM-1, MCP-1 expression is upregulated as measured with Western blot. (^**^ p <0.01, ^***^ p <0.001).

Inverse correlation of ICAM-1 and MCP-1 expressions in a virus infected swine lungs has been reported [20]. To further study this finding, we examined the expression of MCP-1 in several tissues of ICAM-1 knockout mice. Interestingly, we observed the increased expression of MCP-1 in the lung tissue by Western blot analysis (Figure 1A), but MCP-1 expression was not increased in heart and kidney tissues of ICAM-1 knockout mice (Figure 1B). To understand whether ICAM-1 regulates MCP-1 expression, we examined MCP-1 expression in the mouse of monocyte macrophages (RAW264.7 cell) after silencing ICAM-1 with siRNA against ICAM-1 (siRNA-ICAM-1). Transfection of siRNA-ICAM-1 was able to induce downregulation of ICAM-1 expression in the cells by 55.3% (Figure 1C), and at the same time MCP-1 production was increased 59.2% (Figure 1D). These data further confirmed that ICAM-1 negatively regulates MCP-1 expression.

### miRNAs profiles in ICAM-1^-/-^ mouse lungs

**Figure 2:**
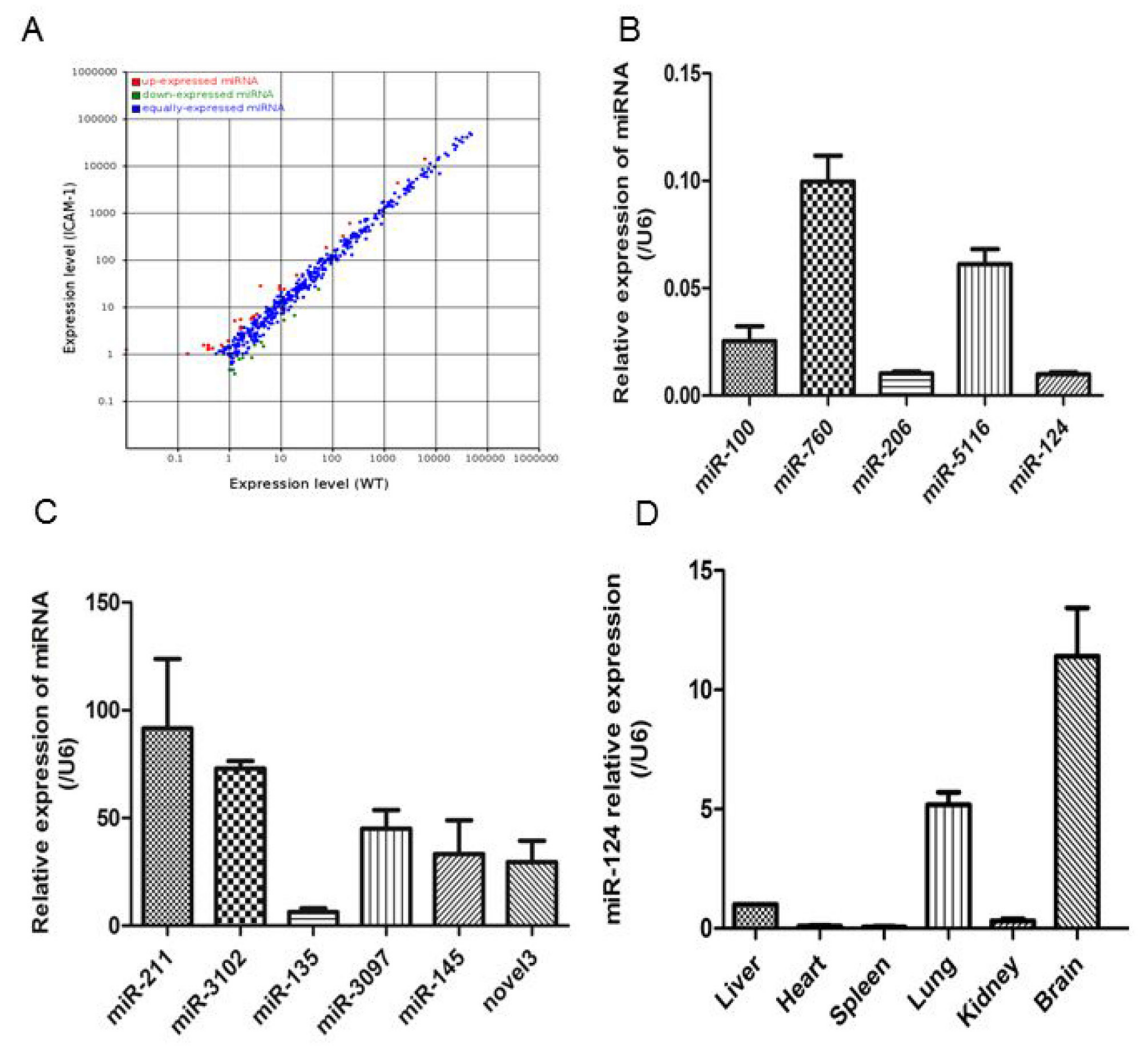
Different miRNA profiles of ICAM-1^-/-^ mouse lungs and wild-type mouse lungs **(A)** Differential expression of known mouse miRNAs in scatter plot. MiRNA profiles in wild-type mouse lungs are distinguished from that in ICAM-1^-/-^ mouse lungs in Table 2. **(B)** and **(C)** Validation of selected microRNA sequence data by real-time qPCR. The relative amounts of each miRNA were normalized to U6 snRNA. **(D)** Analysis of miR-124 relative expression in the wild type mouse tissues by real-time qPCR. The data in graph were shown in mean ± SEM of 3 mice.

To understand how ICAM-1 regulates MCP-1 expression, we analyzed and compared the miRNA expression profiles in the lungs of wild-type or ICAM-1^-/-^ mice. The scatter plot showed a marked difference in several miRNA expression levels (Figure 2A). Among 474 miRNAs analyzed, 29 were upregulated (>2-fold) in ICAM-1 knockout mice lung tissue, and 9 were downregulated compared to wild type mice lung tissue (Table 1). The miRNAs that were reproducibly and reliably altered in the small RNA sequence (P<0.01 and >2-fold change) were considered to be differentially expressed miRNAs and were further assessed quantitatively by real-time qPCR. Such as miR-100, miR-124, miR-206, miR-5116 and miR-760 were significantly downregulated in ICAM-1 knockout mouse lung tissue. Meanwhile miR-135b, miR-145b, miR-211, miR-3097, miR-3102 were significantly upregulated compared to that in wild type mouse lungs. The novel miR-3 was significantly upregulated in ICAM-1 knockout mouse lungs (Figure 2B and 2C).

**Table 1:** The significant difference of miRNAs in WT and ICAM-1^-/-^ group

miR-name	X-std	Y-std	Fold-change (log2 Y/X)	p-value	Sig-lable
mmu-miR-211-5p	0.01	1.2314	6.9441557	1.92E-05	^**^
mmu-miR-135b-5p	4.0329	27.7827	2.7842973	4.82E-57	^**^
mmu-miR-3102-3p	0.1582	1.0005	2.6608997	0.0049306	^**^
mmu-miR-3097-3p	0.3163	1.5392	2.2828152	0.0011502	^**^
mmu-miR-3473e	1.2652	5.0794	2.0052926	2.78E-08	^**^
mmu-miR-145b	0.3954	1.5392	1.9607959	0.0031128	^**^
mmu-miR-448-3p	0.3954	1.3853	1.8088137	0.0080379	^**^
mmu-miR-7010-5p	0.3954	1.3083	1.7263086	0.012748	^*^
mmu-miR-3473b	1.6606	5.3872	1.697831	3.19E-07	^**^
mmu-miR-190a-3p	0.3954	1.2314	1.6389147	0.0200215	^*^
mmu-miR-331-3p	9.4892	27.9366	1.5577981	3.50E-28	^**^
mmu-miR-183-3p	0.4745	1.3083	1.4632134	0.0268781	^*^
mmu-miR-218-1-3p	0.4745	1.3083	1.4632134	0.0268781	^*^
mmu-miR-144-3p	216.8293	594.7493	1.455722	0	^**^
mmu-miR-20b-5p	9.4892	24.1655	1.3485905	4.00E-20	^**^
mmu-miR-33-5p	76.2303	182.242	1.257419	1.40E-126	^**^
mmu-miR-142a-5p	1834.4297	4332.7118	1.2399387	0	^**^
mmu-miR-674-3p	20.1647	46.9458	1.2191642	2.18E-32	^**^
mmu-miR-219a-5p	1.6606	3.7711	1.1832808	0.0011484	^**^
mmu-miR-142a-3p	6184.7753	13945.748	1.1730322	0	^**^
mmu-miR-700-3p	2.8468	6.0799	1.0947065	9.90E-05	^**^
mmu-miR-188-3p	1.6606	3.5402	1.0921263	0.0031495	^**^
mmu-miR-16-2-3p	2.6095	5.5411	1.086399	0.0002229	^**^
mmu-miR-879-5p	3.0049	6.3107	1.0704831	9.79E-05	^**^
mmu-miR-99a-3p	11.4662	23.8577	1.0570676	5.17E-14	^**^
mmu-miR-15b-5p	158.8658	324.1568	1.0288833	1.25E-162	^**^
mmu-miR-338-5p	1.6606	3.3863	1.0280052	0.0059983	^**^
mmu-miR-1264-3p	0.9489	1.924	1.0197808	0.0411419	^*^
mmu-miR-15a-3p	3.4794	7.0034	1.009217	9.12E-05	^**^
mmu-miR-760-3p	1.2652	0.3848	-1.7171848	0.0142121	^*^
mmu-miR-100-3p	2.7677	0.8466	-1.7089352	0.000243	^**^
mmu-miR-124-3p	4.5865	1.4622	-1.649253	3.66E-06	^**^
mmu-miR-206-3p	18.3459	6.6186	-1.4708597	1.08E-17	^**^
mmu-miR-5116	1.1862	0.4618	-1.3610072	0.0452139	^*^
mmu-miR-184-3p	4.1911	1.7701	-1.2434981	0.0003454	^**^
mmu-miR-122-5p	54.0096	23.9347	-1.1741121	4.30E-35	^**^

mmu-miR-193b-3p	11.4662	5.2333	-1.1315945	3.44E-08	^**^
mmu-miR-1839-3p	1.8188	0.8466	-1.1032345	0.0336701	^*^
mmu-miR-1249-3p	3.084	1.5392	-1.0026221	0.0100169	^*^

**Table 2:** Upregulated and downregulated miRNAs in ICAM-1-/- group

miRNA	Normal (2^ΔCT^)	ICAM-1^-/-^ (2^ΔCT^)	Fold-change	p-value
mmu-miR-100-3p	2.7677	0.8466	-1.7089352	0.000243
mmu-miR-124-3p	4.5865	1.4622	-1.649253	3.66E-06
mmu-miR-206-3p	18.3459	6.6186	-1.4708597	1.08E-17
mmu-miR-5116	1.1862	0.4618	-1.3610072	0.0452139
mmu-miR-760-3p	1.2652	0.3848	-1.7171848	0.0142121
mmu-miR-135b-5p	4.0329	27.7827	2.7842973	4.82E-57
mmu-miR-145b	0.3954	1.5392	1.9607959	0.0031128
mmu-miR-211-5p	0.01	1.2314	6.9441557	1.92E-05
mmu-miR-3097-3p	0.3163	1.5392	2.2828152	0.0011502
mmu-miR-3102-3p	0.1582	1.0005	2.6608997	0.0049306
novel miR-3	11.6243	31.9385	1.45815272	3.61E-29

In the ICAM-1 knockout mouse lungs, the miR-124 expression was significantly reduced. It was reported before that miRNA-124 specifically expressed in brain tissue [21], we analyzed miR-124 expression in wild-type mouse brain, heart, liver, spleen, lung, and kidney tissues, and the results showed that miR-124 expression is abundant in mouse brain, followed by lung (Figure 2D).

### MiR-124 directly binds and downregulates MCP-1 expression

**Figure 3:**
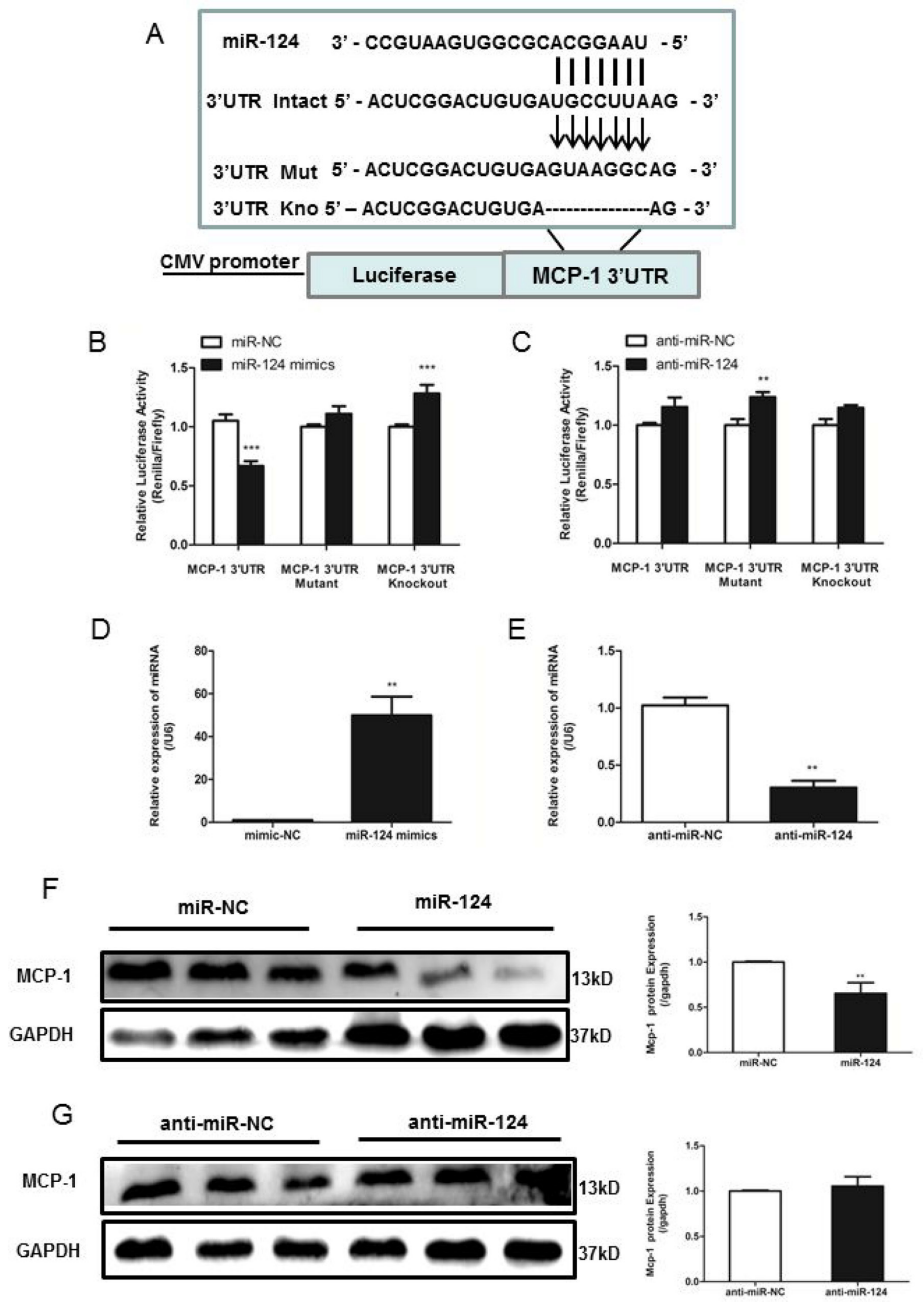
MiR-124 regulates monocyte chemotactic protein 1 (MCP-1) mRNA and protein expression via direct binding to the 3’UTR of MCP-1 **(A)** The reporter constructs used in this study, psicheck2-MCP-1 3’UTR, psicheck2-Mutant MCP-1 3’UTR, the seed region is replaced random mutated and psicheck2-Knockout MCP-1 3’UTR, seed region is deleted. **(B)** and **(C)** RAW264.7 cells were co-transfected with psicheck2-MCP-1 3’UTR or psicheck2-Mutant MCP-1 3’UTR/ psicheck2-Knockout MCP-1 3’UTR together with miR-124 mimics or the mimics control, negative control (anti-miR ctrl) and control inhibitor (anti-miR-124 mimics) as indicated. After 24h, firefly luciferase activity was determined and normalized to renilla luciferase activity. Similar results were obtained in three independent experiments. The data in graph were shown in mean ± SEM of 3 different transfections. **(D)** and **(E)** Real-time qPCR analysis of expression levels of miR-124 in RAW264.7 after transfected with mimic-NC, miR-124 mimics, inhibitor-NC and miR-124 inhibitor. **(F)** and **(G)** RAW264.7 cells were transfected with miR-124 mimic or the mimic control, inhibitor-NC or miR-124 inhibitor for 48h, and then MCP-1 protein levels were measured with Western blotting. (^**^ p <0.01, ^***^ p <0.001).

Small RNA sequencing analysis by software showed that miR-124 might target MCP-1. MiRNAs regulate gene expression by binding to target sites of mRNAs and causing their degradation or translational repression. We then determined whether miR-124 was able to target MCP-1, and a dual-luciferase reporter assay was performed. The potential binding site of miR-124 in the 3’UTR of the mouse MCP-1 mRNA was determined by miRanda. To test the specific regulation of MCP-1 via the predicted binding sites, we subcloned the MCP-1 3’UTR sequence into a psiCheck2 Dual-Luciferase miRNA target Expression Vector (Figure 3A). A mutant of MCP-1 3’UTR, random mutated bases in the seed region or another mutant with deletion seed region was also subcloned. After co-transfection of miR-124 mimic and the wild-type pLuc-MCP-1 into RAW264.7 cells, luciferase activity was performed. The results showed that miR-124 mimic significantly inhibited the luciferase activity of pLuc-MCP-1 (Figure 3B and 3C). And mutation of the miR-124 target site abolished miR-124 mimic effects, indicating miR-124 specifically targets the MCP-1 3’UTR.

Next, we transiently transfected RAW264.7 cells with miR-124 mimic, miR-124 inhibitor, or control vector, respectively. Mature miR-124 expression in transfected cells was confirmed by real-time qPCR (Figure 3D and 3E). Transfection of RAW264.7 cells with miR-124 mimic decreased MCP-1 expression analyzed by Western blot analysis, whereas anti-miR-124 treatment resulted in increased MCP-1 expression (Figure 3F and 3G).

### ICAM-1 depletion increases MCP-1 expression by suppressing miR-124 levels

**Figure 4:**
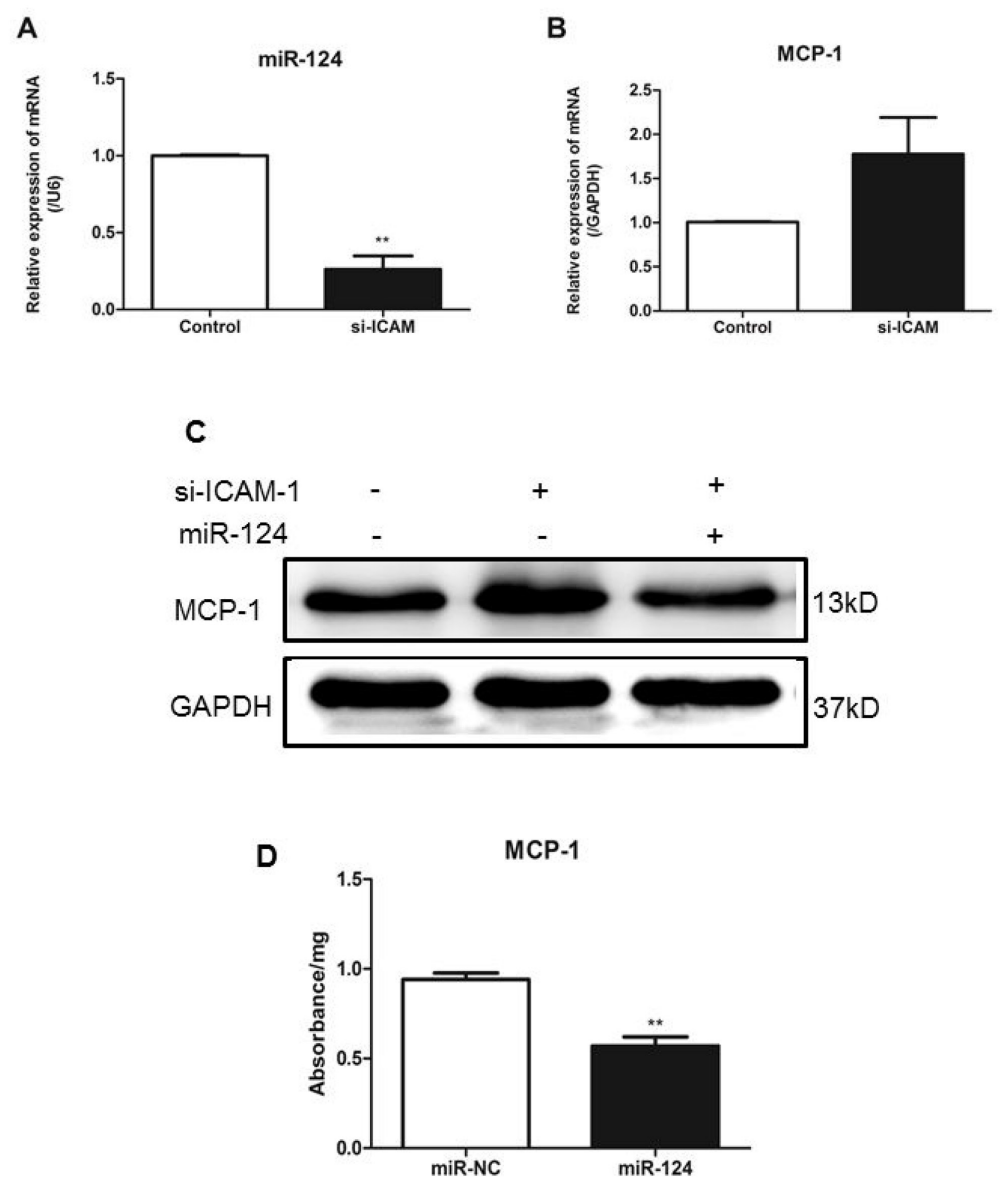
ICAM-1depletion attenuates miR-124 expression and promotes MCP-1 production in macrophages Downregulation of miR-124 **(A)** and upregulation of MCP-1 **(B)** in RAW264.7 cells transfected with siRNA against ICAM-1. Twenty-four h after transfection, expressions were measured with real-time qPCR. The data in graph were shown in mean ± SEM of 3 different transfections. **(C)** MCP-1 protein levels were upregulated when ICAM-1 was downregulated. RAW264.7 cells were co-transfected with siRNA-ICAM-1 and miR-124 mimics, MCP-1 protein levels were measured with Western blot analysis 48h later. **(D)** MCP-1 protein expression was determined by ELISA. (^**^ p <0.01).

To determine the effects of downregulation of ICAM-1 on expression of miR-124 and MCP-1, RAW264.7 cells were transfected with siRNA-ICAM-1 and expression of miR-124 was measured by real-time qPCR. ICAM-1 downregulation resulted in reduced expression of miR-124 (Figure 4A). This observation was consistent with our previous observation in the ICAM-1 knockout mouse lungs. In addition, ICAM-1 knockdown caused MCP-1 production increased (Figure 4B).

Next, we co-transfected RAW264.7 cells with miR-124 mimics and siRNA-ICAM-1 and then measured MCP-1 expression. Co-transfection of miR-124 mimics and siRNA-ICAM-1 resulted in attenuation of the increased MCP-1 expression induced by downregulation of ICAM-1 (Figure 4C). Furthermore, we using ELISA examined the protein levels of MCP-1 in the macrophage lysates (Figure 4D). These results suggest that miR-124 blunts MCP-1 production induced by ICAM-1 deficiency.

### Sp1 is required for miR-124 transcription

**Figure 5:**
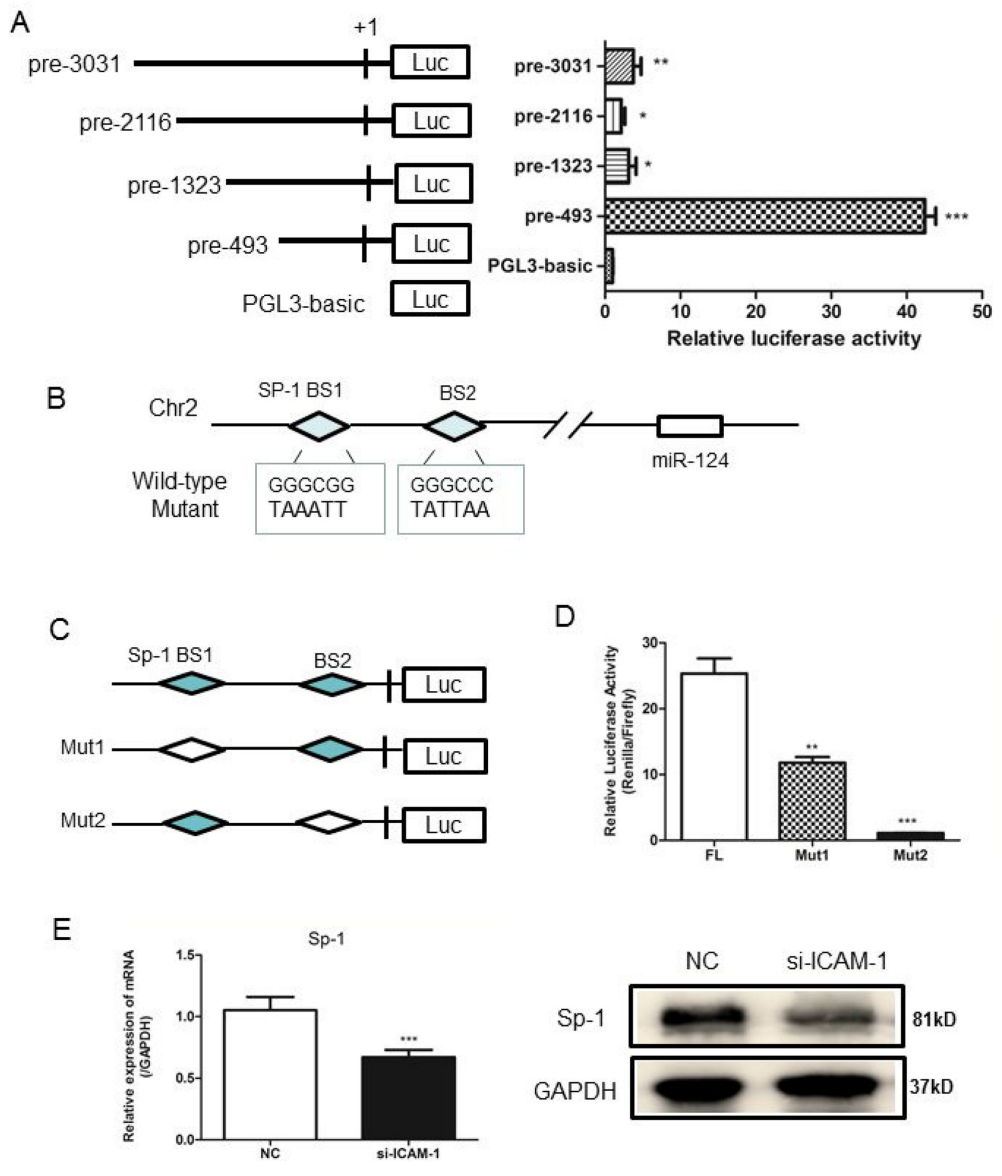
Analysis of the miR-124 promoter and transcription factor Sp1 binds to miR-124 promoter **(A)** RAW264.7 cells were co-transfected with pRL-TK and a miR-124 promoter reporter plasmid containing various lengths of the miR-124 promoter. The first nucleotide of mature pre-miR-124 is assigned +1. Right panel shows the relative luciferase activities of these plasmids. Luciferase activity of the mock transfected empty vector pGL3-Basic was used for normalization. The data in graph were shown in mean ± SEM of 3 different transfections. **(B)** Schematic diagram of miR-124 genomic location in mouse chromosome 2. The promoter region from pre-197 to pre-27 was used for analysis of potential transcription factor binding sites. Two Sp1 binding sites are indicated as diamonds. The sequences of mutated sites are shown under the locus diagram. **(C)** Schematic representation of deletion mutants 1 and 2 in the full-length miR-124 promoter. **(D)** RAW264.7 cells were co-transfected with the above promoter reporter constructs and pRL-TK. Post-transfection for 36h, samples were collected and analyzed for dual luciferase activity. **(E)** Real-time qPCR and Western blot analysis of the expression of Sp1 after transfection with ICAM-1 siRNA and negative control. (^*^ p < 0.05, ^**^ p <0.01, ^***^ p <0.001).

To understand how ICAM-1 regulates miR-124, we then determined the transcriptional regulation of miR-124. Genomic information and chromosomal location of miR-124 were obtained from miRBase and its potential promoter was analyzed. A schematic representation of the miR-124 promoter was shown in Figure 5A. To define the boundaries of the minimal promoter region, an approximately 3 kb DNA fragment containing non-coding sequences upstream of the first nucleotide site of pre-miR-124 promoter was cloned and then transfected into RAW264.7 cells to analyze the promoter activity. DNAs containing different length of miR-124 promoter sequence, such as pre-3031, pre-2116, pre-1323 or pre-493, were subcloned into the reporter plasmid for promoter activity analysis. The highest luciferase activity was produced by reporter pre-493(Figure 5A), suggesting that the pre-493 contained the intact promoter region. This region was chosen for further analysis. And the others region may contain a negative-acting elements. Bioinformatics analyses of the selected promoter region using JASPAR and CONSITE software predicted Sp1 (specificity protein 1) as transcription factors. The two binding sites of Sp1 on the selected promoter region were shown in Figure 5B. To determine whether Sp1 regulates expression of miR-124 in RAW264.7 cells, a two truncated promoter constructs including full-length, deletion mut1 (BS1) and mut2 (BS2) were generated (Figure 5C). Compared with the full-length promoter construct, deletion of one of Sp1 binding site resulted in the decreased luciferase activity (Figure 5D). Both real-time qPCR and Western blot analyses showed that expression of Sp1 was significantly reduced at mRNA and protein levels when ICAM-1 was downregulated by siRNA-ICAM-1 (Figure 5E).

### Differential regulation of M1/M2 macrophage polarization by ICAM-1 and miR-124

**Figure 6:**
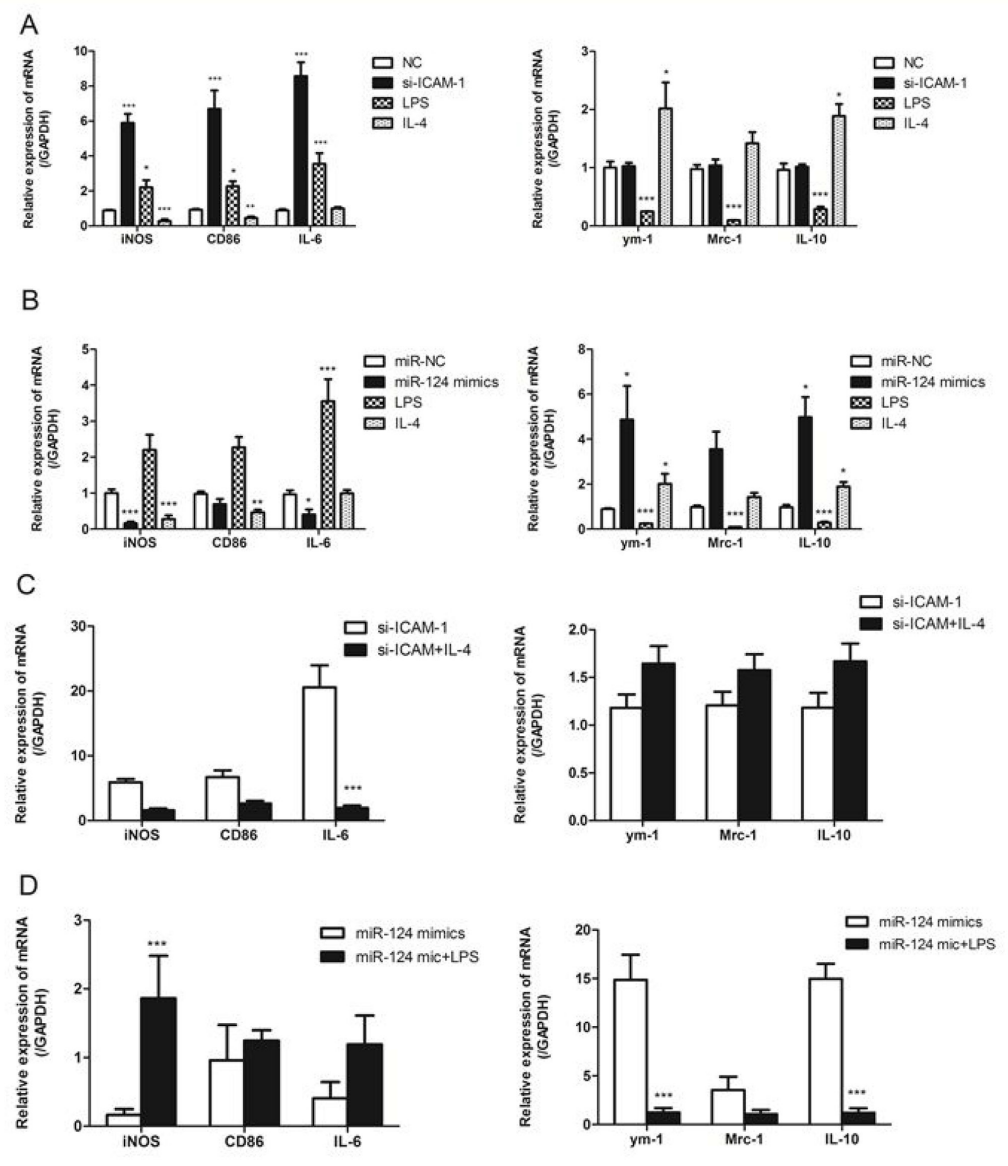
ICAM-1 and miR-124 induce different macrophage polarizations **(A)** Real-time qPCR analysis of the mRNA expression of M1 marker (iNOS, CD86, IL-6) and M2 (ym-1, Mrc-1, IL-10) markers in ICAM-1 siRNA or negative control transfected with RAW264.7 cells. RAW264.7 cells treated with for 24h. **(B)** RAW264.7 cells were transfected with miR-124 mimics or negative control, LPS (2ug/ml) or IL-4 (20ng/ml), mRNA expressions of M1 and M2 markers were analyzed with real-time qPCR. **(C)** The mRNA expressions of M1 and M2 markers in RAW264.7 cells transfected with siRNA-ICAM-1 for 24h in the presence or absence of IL-4. **(D)** The mRNA expressions of M1 and M2 markers in RAW264.7 cells transfected with miR-124 mimics for 24h in the presence or absence of LPS. (^*^ p < 0.05, ^**^ p <0.01, ^***^ p <0.001).

It is known that macrophage M1/M2 polarization was able to induce by the treatment of macrophages with LPS or IL-4 [22]. Treatment of RAW264.7 cells with LPS resulted in upregulation of the M1 markers such as iNOS, CD86, IL-6 and downregulation of the M2 markers including ym-1, Mrc-1 and IL-10. Meanwhile, treatment of RAW264.7 cells with IL-4 resulted in upregulation of M2 markers and downregulation of M1 markers. We then investigated whether ICAM-1 or miR-124 expression affected the macrophage polarization. To test the role of ICAM-1 in macrophage polarization, the effects of downregulation of ICAM-1 were examined. Downregulation of ICAM-1 by siRNA-ICAM-1 resulted in M1 polarization, as shown the M1 marker expression (Figure 6A). To determine the effects of miR-124 on the macrophage polarization, miR-124 was overexpressed in RAW264.7 and the M1 and M2 markers were measured with real-time qPCR. Overexpression of miR-124 also resulted in M2 polarization as downregulation of M1 markers and upregulation of the M2 markers (Figure 6B).

Next we treated siRNA-ICAM-1 transfected RAW264.7 cells with IL-4 and measured M1 and M2 markers. We found that decreased expression of M1 markers iNOS, CD86 and IL-6 and increased expression of M2 markers ym-1, Mrc-1 and IL-10 when compared to control (Figure 6C). We further investigated whether miR-124 expression affects expression of the M2 markers of the polarized macrophages. We performed the M1 polarization of the macrophages by LPS in the presence of miR-124 mimics or negative control. The miR-124 had a little effect on the expression of M1 and M2 markers in the LPS treated RAW264.7 cells (Figure 6D). These data suggest that the polarized macrophage induced by siRNA-ICAM-1 or miR-124 are still able to response the stimuli LPS or IL-4.

### ICAM-1 regulation of MCP-1 expression via miR-124 in porcine alveolar macrophages

**Figure 7:**
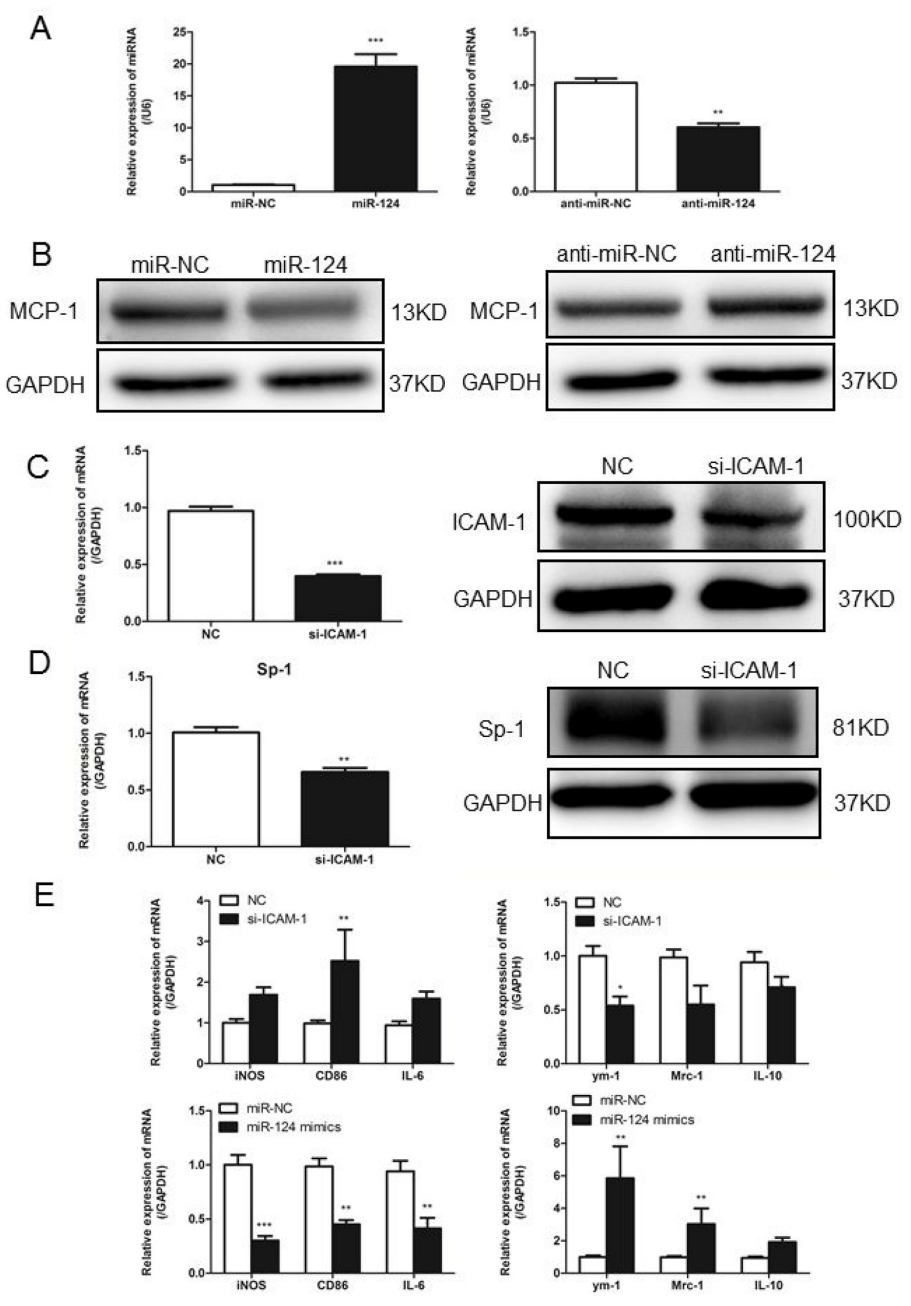
In primary macrophages confirm ICAM-1 regulation of MCP-1 expression via miR124 **(A)** Real-time qPCR analysis of expression levels of miR-124 in porcine alveolar macrophages (primary macrophages) after transfected with mimic-NC, miR-124 mimics, inhibitor-NC and miR-124 inhibitor. **(B)** PAM cells were transfected with miR-124 mimic or mimic control, inhibitor-NC or miR-124 inhibitor for 48h, MCP-1 protein levels were measured with Western blotting. **(C)** In PAM cells transfected with siRNA-ICAM-1, knockdown of ICAM-1 was measured with Western blot. **(D)** Real-time qPCR and Western blot analysis of the expression of Sp1 after transfection with ICAM-1 siRNA and negative control. **(E)** The mRNA expressions of M1 and M2 markers in PAM cells transfected with siRNA-ICAM-1 or miR-124 mimics for 24h. (^*^ p < 0.05, ^**^ p <0.01, ^***^ p <0.001).

To confirm our findings regarding ICAM-1 regulation of MCP-1 expression via miR-124 in primary macrophages, we isolated the porcine alveolar macrophages (PAM) from porcine lung tissue. We transiently transfected PAM cells with miR-124 mimic or miR-124 inhibitor. Mature miR-124 expression in transfected cells was confirmed by real-time qPCR (Figure 7A). Transfection of PAM cells with miR-124 mimic decreased MCP-1 expression analyzed by Western blot analysis, anti-miR-124 transfection resulted in increased MCP-1 expression (Figure 7B). Transfection of PAM cells with siRNA-ICAM-1 was able to induce downregulation of ICAM-1 expression (Figure 7C). Real-time qPCR and Western blot analyses showed that expression of Sp1 was reduced at mRNA and protein levels by siRNA-ICAM-1 (Figure 7D). Furthermore, we measured the expression of M1 and M2 markers in siRNA-ICAM-1 or miR-124 mimic transfected PAM cells. Both downregulation of ICAM-1 and overexpression of miR-124 were able to induce M2 polarization in porcine alveolar macrophages (Figure 7E), further demonstrating the regulatory roles of ICAM-1 and miR-124 in macrophage polarization.

### Exogenous administration of miR-124 protects against acute lung injury

**Figure 8:**
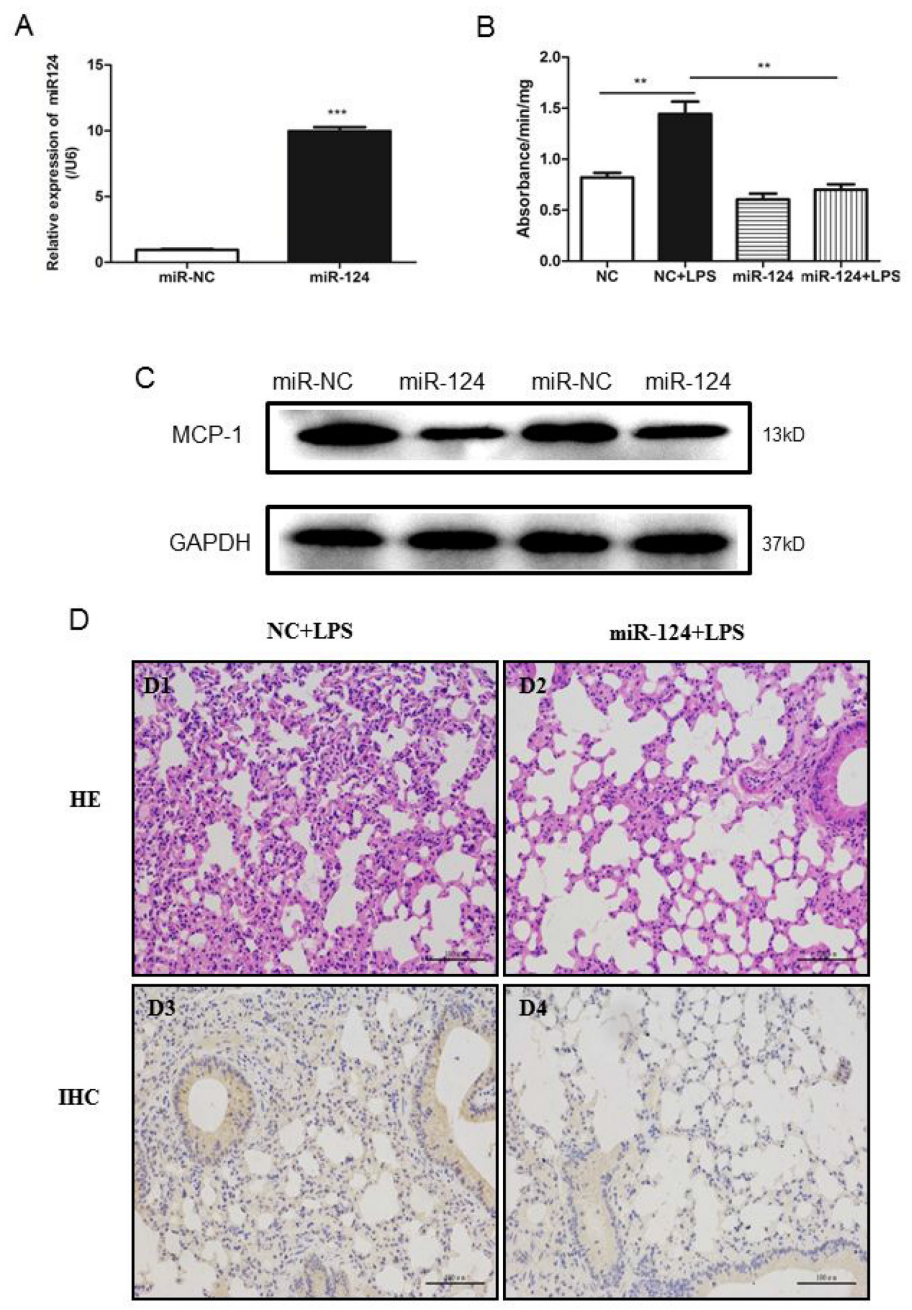
MiR-124 protects against lung injury in mice **(A)** Real-time qPCR detection of miR-124 in the lung tissues of C57BL/6 mice 48 hours after tail-vein injection with miR-124 mimics and negative control. **(B)** Forty-eight h after tail-vein injection of negative control and miR-124 mimics, mice were treated with tail-vein LPS (10mg/kg) or PBS injection. The lungs were collected 6 h later and MPO activity was assayed in lung tissue. The data in graph were shown in mean ± SEM of 3 mice. (n=3 for each groups ^**^ p <0.01, ^***^ p <0.001). **(C)** Western blots analysis of expression of MCP-1 in mouse lungs. **(D)** Lung injury HE stain and MCP-1immunohistochemistry in lungs from mice were treated with intravenous injection LPS and miR-124 mimics.

To understand the role of miR-124 *in vivo*, we examined the effects of miR-124 on lung injury using a mouse model. Intravenous injection of miR-124 mimics and liposome mixture was performed to overexpress miR-124 in mouse lung (Figure 8A). The lung injury was introduced by intravenous injection of LPS and measured with MPO activity. As shown in Figure 8B, LPS induced high myeloperoxidase (MPO) activity was remarkably attenuated by miR-124, suggesting the protective role of miR-124 agaist lung injury. We also examined the effects of miR-124 on the expression of MCP-1. In the mouse lungs, MCP-1 expression was decreased in the miR-124 mimics group compared with the negative control group (Figure 8C). As shown in Figure 8D, intravenous injection LPS showed markedly alveolar septal thickening, erythrocyte exudation and inflammatory cell infiltration in the lungs (Figure 8D1). Moreover, LPS induced lung morphological damage was remarkably attenuated by miR-124 (Figure 8D2). Intravenous injection of miR-124 mimics also downregulated MCP-1 expression in mouse lungs, as measured by immunohistochemistry analysis (Figure 8D3, D4).

In addition, we also used real-time qPCR to determine the mRNA expression of ICAM-1, miR-124 and MCP-1 in a swine lung injury caused by infection of porcine reproductive and respiratory syndrome virus (PRRSV). As shown in Supplementary Figure 1, both ICAM-1 and miR-124 mRNA were significantly upregulated in infected swine lungs, the MCP-1 mRNA expression downregulated in swine lungs (Supplementary Figure 1A). Western blot analysis further confirmed increased ICAM-1 expression and decreased MCP-1 expression in the PRRSV-infected swine lungs (Supplementary Figure 1B). Taken together, ICAM-1 and miR-124 are regulating MCP-1 production in inflammatory lungs.

## DISCUSSION

ICAM-1 is important for leukocyte firm adhesion to endothelial cells during inflammatory tissue injury, and initials intracellular signaling required for leukocyte trans-endothelial migration [23]. Inflammatory factors, such as TNF-ɑ, also stimulates ICAM-1 expression in mouse macrophages [8]. A recent report suggests that ICAM-1 regulates macrophage polarization [11]. Here, we reported a novel role of ICAM-1 in the downregulation of miR-124 mediated expression of MCP-1, a key regulator molecule in monocytes recruitment, in both lungs and macrophages.

We first observed the increased MCP-1 expression in the ICAM-1 knockout mouse lungs. MCP-1expression in heart and kidney tissue was down-regulated. The result suggests that ICAM-1 specifically inhibit MCP-1 production in lung tissue. We reported an inverse relationship between ICAM-1 and MCP-1 expression in a lung injury [20]. In addition, Downregulation of ICAM-1 with siRNA in mouse macrophages also presented increased MCP-1 expression, suggesting that ICAM-1 regulates MCP-1 production in macrophages. In the effort to understand the mechanism of this regulation, we then analyzed the miRNA profile and identified miR-124 might be the potential candidate to medicate the regulation of ICAM-1 on MCP-1 production based on those observations: (1) miR-124 expression is lower in ICAM-1 knockout mouse lungs compared to wild-type ones, (2) miR-124 is highly expressed in lung and brain, and no MCP-1 production in heart and kidney tissues from ICAM-1knockout mice. Previous studies from others on pulmonary hypertension fibroblasts and heumatoid arthritis synoviocytes showed that MCP-1 production is under the direct control of miR-124 [18, 24]. Furthermore, our data also show that miR-124 directly targets the 3’UTR of MCP-1. Luciferase reporter assay demonstrated that miR-124 specially suppressed the reporter activity driven by the 3’UTR MCP-1 mRNA. Overexpression of miR-124 in RAW264.7 cells suppressed the expression of MCP-1. These results indicate that miR-124 mediates the ICAM-1 regulated MCP-1 production in macrophages.

To understand how ICAM-1 regulates miR-124, our study next focused on the transcriptional regulation of miR-124. Analysis of miR-124 gene promoter and activity assay suggest that the transcriptional factor Sp1 binds to the miR-124 promoter and ICAM-1 knockdown dramatically decreases Sp1 expression in cultured macrophages. Further study confirmed that down-regulation of Sp1 by ICAM-1 knockdown contributed to low miR-124 expression in macrophage. Recent reports also demonstrated that Sp1 promotes other miRNA gene expression such as miR-19a, and Sp1 was also regarded as an upstream factor of miR-19a mediated down-regulation of RHOB and promotion of pancreatic cancer [25]. Thus, Sp1 may regulate the processing of multiple miRNAs. Further studies are needed to determine whether other transcription factors and Sp1 may collaborate in the transcriptional regulation of miR-124 in ICAM-1 knockdown macrophages.

To understand the biological roles of ICAM-1 regulating miR-124, we studied the effects of miR-124 or ICAM-1 on macrophage polarization. It is well known that LPS and IL-4 induce M1 or M2 macrophage respectively [26-28]. In this study, by measuring the expression of M1 markers and M2 markers, we also observed that LPS stimulated M1 macrophage polarization, IL-4 stimulation induced M2 macrophage polarization. ICAM-1 depletion by siRNA in RAW264.7 cells increased the expression of pro-inflammatory factors including iNOS, CD86 and IL-6, suggesting that ICAM-1 moderates macrophage polarization. Our results are not exactly consistent with a previous report, in which ICAM-1 was found to suppress M2 macrophage polarization and inhibit metastatic tumor progression [11]. Nevertheless, both studies suggest the ICAM-1 is able to regulate the macrophage polarization and this regulation may be affected by other factors. Another related issue is MCP-1 induced macrophage polarization. MCP-1 is able to induce both pro-inflammatory and anti-inflammatory effects on macrophages, suggesting the complicated role of MCP-1 in macrophage polarization [29]. Blockage of MCP-1 with antibody increased gene expression associated with M1 polarization [29]; however, macrophages with MCP-1 knockout are still able to produce M2 associated factors in response to IL-4 [30]. In addition, MCP-1 is a marker of M1 polarization [26], which is consistent with our results. MCP-1 may have different effects on polarization depend on the local availability of pro-inflammatory factors. It is likely that miR-124 may regulate the expression of other inflammatory factors and MCP-1 may work together with them to decide the direction of macrophage polarization. Future research will address these possibilities to understand the molecular mechanism of the complexity of MCP-1induced macrophage polarization.

Several lines of evidence indicate that miRNAs as important regulators for gene expression in macrophages. miR-33 plays a dual role in deciding macrophage polarity and its conversion to foam cell [31]. Meanwhile, miR-33 as a suppressor of MCP-1 expression, providing a potential mechanism of macrophage infiltration in chronic inflammation [32]. MiR-223 was reported to differentially express during macrophage polarization, elevation in LPS treated macrophages, and downregulation in IL-4 treated BMEMs [33]. MiR-146a significantly decreased expression of pro-inflammatory cytokines including iNOS and TNF-α in M1 macrophage, while increased expression of M2 marker genes (Arg1 and CD206). MiR-146 modulates macrophage polarization by inhibiting Notch1 pathway [34]. MiR-124 plays a key role in inhibiting macrophage activation and promoting microglial differentiation by inhibiting the transcription factors C/EBP-α and PU.1 [35, 36]. In our study transfection of macrophage with miR-124 mimics increased the expression of anti-inflammatory cytokines including ym-1, Mrc-1 and IL-10, and caused no significant changes of M1 macrophage markers, suggesting that in contrast to ICAM-1 knockdown, miR-124 mimics promotes the M2 macrophage polarization. Taken with the regulation of ICAM-1 on the expression of miR-124, our results also suggest that the regulation of ICAM-1 on macrophage polarization is likely through miR-124.

The close relationship of miR-124 with inflammation was also revealed in a porcine lung injury caused by PRRSV infection. In porcine lung infected by PRRSV, our previous study shows that inversely relationship of ICAM-1 and MCP-1 [20]. Analysis of miR-124 expression in infected porcine lungs suggests that there are positive correlation of ICAM-1 and miR-124. To understand whether miR-124 is able to modulate inflammation *in vivo*, we have injected miR-124 in the mice treated with LPS. LPS induced MPO activity in the mouse lungs was greatly decreased by co-injection of miR-124. The protection role of miR-124 in the mouse lung injury model clearly suggests that miR-124 is able to regulate inflammation. It is well known that miRNAs modulates inflammatory and immune mediated diseases by regulating their cellular and molecular targets [33, 37, 38]. Although our studies suggest miR-124 modulates the inflammation in macrophage and mouse lungs by targeting to MCP-1, other miR-124 targets may also play some roles. Recent reports suggest that miR-124 may target USP2 and USP14, then in turn negatively regulates LPS-induced TNF-α production in mouse macrophage *in vivo* and vitro [28]. Nevertheless, our studies also suggest that miR-124 may have potential therapeutic function against inflammation.

In summary, our studies demonstrated that ICAM-1 regulates both miR-124 and MCP-1 expression in macrophages. ICAM-1 stimulates miR-124 expression by increasing Sp1 activation, and Sp1 in turn binds to the miR-124 promoter and transactivates its expression. ICAM-1 upregulated miR-124 suppresses MCP-1 production via direct binding to the 3’UTR of MCP-1. ICAM-1 deficiency induces M1 macrophage polarization and overexpression of miR-124 promoted M2 macrophage polarization. Furthermore, overexpression of miR-124 protects mice against LPS-induced acute lung injury. Our results suggest a novel role of ICAM-1 in the regulation of inflammation and raise a possibility that targeting ICAM-1/miR-124/MCP-1 axis in modulating inflammation during acute lung injury.

## MATERIALS AND METHODS

### Ethics statement section

All animal procedures were approved by the Animal Care and Use Committee of Hubei Province, China, in accordance with guidelines developed by the China Council on Animal Care and Protocol.

### Mice

C57BL/6 mice wild type (WT) and knockout strain (ICAM^-/-^) used for MCP-1 analysis and miRNA sequencing were purchased from the Jackson laboratory and maintained in University of Illinois at Chicago. Mice weight 20∼30g and 10∼12 weeks of age were used. C57BL/6 mice used in miR-124 injection were purchased from the Disease Prevention and Control Center (Wuhan, China). Mice weight 18∼25g and 7∼8 weeks.

### Reagents

Mice mmu-miR-124 dsRNA mimics, ssRNA inhibitor and control oligonucleotides were synthesized by GenePharma (Shanghai, China). The miRNA nucleic acid sequences are listed in Table 3. The commercial of ICAM-1 siRNA (sc-29355), antibodies ICAM-1 and MCP-1 were purchased from Santa Cruz. Antibodies against Sp1 and GAPDH were purchased from Proteintech Group (Wuhan, China). LPS was purchased from Sigma (Shanghai, China) and recombinant murine IL-4 from Peprotech (Beijing, China).

**Table 3:** Nucleic acid sequences for miR-124 mimics and inhibitor

miRNA	Forward (5’ to 3’)
miR-124 mimics	SENSE: UAAGGCACGCGGUGAAUGCC
	ANTI-SENSE: CAUUCACCGCGUGCCUUAUU
mimics NC	SENSE: UUCUCCGAACGUGUCACGUTT
	ANTI-SENSE: ACGUGACACGUUCGGAGAATT
miR-124 inhibitor	UGGCAUUCACCGCGUGCCUUA
inhibitor NC	CAGUACUUUUGUGUGUAGUACAA

### Cell culture and treatment

Mouse RAW264.7 macrophages were purchased from ATCC (Manassas, VA), and maintained in DMEM medium supplemented with 10% FBS. The cells cultured at 37°C in 5% CO_2_. RAW264.7 cells were plated in 12-well plates (2×10^5^ cell/well), grown to 80% confluent, and then transfected with plasmids or RNAs using Lipofectamine 2000 from Invitrogen (Shanghai, China).

The 3-week-old large white piglets were obtained and tested negative for both porcine circovirus type 2 (PCV2) and porcine reproductive and respiratory syndrome virus (PRRSV). The piglets were sacrificed and lungs were removed. The sufficient numbers of porcine alveolar macrophages were obtained by bronchoalveolar lavage, and purified alveolar macrophages were obtained *in vitro* by adherent selection.

### Small RNA sequence

Total RNA was extracted using miRNeasy Mini Kit from QIAGEN (Guangzhou China) according to the manufacturer’s protocol. Total RNA integrity was measured on an Agilent 2100 Bioanalyzer system (Agilent) for quality control. RNA fragments with 16∼35nt were excised, purified from a PAGE gel, and ligated with 5’and 3’adaptors using T4 RNA ligase. Reverse transcription followed by PCR was used to create cDNA constructs based on the small RNA ligated with 3’and 5’adapters. Subsequently, the amplified cDNA constructs were purified from agarose gel, in preparation for sequencing analysis using the Illumina Genome Analyzer (San Diego, CA, USA) according to the manufacturer’s instructions. The data were then processed using Illumina Genome Analyzer Pipeline software and then submitted to data filtration. Clean reads were obtained after filtering low-quality reads and trimming the adaptor sequences. All of the clean reads were initially searched against miRBase 19 to identify known mouse miRNAs. Unmappable reads subsequently were annotated and classified by reference to non-coding RNAs in the Ensemble, piRNAand Rfam databases. The mappable sequences were achieved and used for further analysis. Meantime, many unannotated sequences that cannot match any above databases were analyzed by miRDeep to predict novel miRNA candidates.

### Real-Time qPCR

Total RNA was extracted with Trizol from Invitrogen (Shanghai, China) and 1ug RNA was used for cDNA synthesis with a first-strand cDNA synthesis kit from GeneMark (Shanghai, China). Quantitative real-time PCR analysis was performed using a LightCycler 96 Real-Time PCR System and SYBR Green PCR master mix both from GeneMark (Shanghai, China). Data were normalized to the level of GAPDH expression in each sample. Primers used are listed in Table 4.

**Table 4:** Sequence of primer for RT-PCR

Primer	Forward (5’ to 3’)	Reverse (5’ to 3’)
mus-GAPDH	CTTCCGTGTTCCTACCC	GCCCAAGATGCCCTTCA
sus-GAPDH	CTGCCGCCTGGAGAAACCT	GCTGTAGCCAAATTCATTGTCG
mus -MCP-1	CCTCGAGAGCCTGACTCCACCC	ATTTGCGGCCGCTTCAATAGTTAC
sus-MCP-1	ccagcagcaagtgtcctaaag	ttttcttgtccaggtggcttat
sus-ICAM-1	cagtgttgcctgtgatggaaa	cttcagtcttgtgccagtgagtct
mus-iNOS	CCCTTCCGAAGTTTCTGGCAGCAGC	CCAAAGCCACGAGGCTCTGACAGCC
mus-CD86	TCAGTCAGGATGGGAGTGGTA	ATCCAAGAGCCATTCCTACCT
mus-IL-6	ATGGCAATTCTGATTGTATG	GACTCTGGCTTTGTCTTTCT
mus-ym-1	TGGAGGATGGAAGTTTGGAC	GAGTAGCAGCCTTGGAATGT
mus -Mrc-1	CATGAGGCTTCTCTTGCTTCTG	TTGCCGTCTGAACTGAGATGG
mus -IL-10	ACAACATACTGCTAACCGACTC	CACTCTTCACCTGCTCCACT

To quantify mature miRNA expression, a commercial miRcute miRNA isolation kit, miRcute miRNA First-Strand cDNA Synthesisi Kit and miRcute miRNA qPCR detection kit from Tiangen (Beijing, China). Briefly, 1ug total RNA was used as the template and reverse transcribed using poly(A) polymerase and specific RT primer. Amplification was performed for 30min at 37°C and 60 min at 37°C, followed by 35 cycles of 95°C for 10s, 58°C for 10s, and 72°C for 30s. The relative expression of miRNAs was normalized to that of internal control U6 within each sample using the 2-^∆∆Ct^ method. Primers used are listed in Table 5. All constructs were verified with sequencing.

**Table 5:** Sequence of primer for RT-PCR miRNA reverse transcription and RT-PCR

Primer	Sequence (5’ to 3’)
mmu-miR-211-5p	TTCCCTTTGTCATCCTTTGCCT
mmu-miR-3102-3p	CTCTACTCCCTGCCCCAGCCA
mmu-miR-135b-5p	TATGGCTTTTCATTCCTATGTGA
mmu-miR-3097-3p	CTCAGACCTTTCTACCTGTCAG
mmu-miR-145b	GTCCAGTTTTCCCAGGAGACT
Novel-miR-3	AGTGTTTCCTACTTTATGGATGA
mmu-miR-100-3p	ACAAGCTTGTGTCTATAGGTAT
mmu-miR-760-3p	CGGCTCTGGGTCTGTGGGGA
mmu-miR-206-3p	TGGAATGTAAGGAAGTGTGTGG
mmu-miR-5116	TTTGATAGGAACCCCGCCTGA
mmu-miR-124-3p	TAAGGCACGCGGTGAATGCC
Universal Reverse	GTGCAGGGTCCGAGGT
U6-Forward	CTCGCTTCGGCAGCACA
U6 Universal Reverse	AACGCTTCACGAATTTGCGT

### Constructs and plasmids

The psiCheck-2 Dual-Luciferase Reporter (Promega, Shanghai, China) harboring the 3’UTR of MCP-1 was inserted into the *XhoI* and *NotI* restriction sites 3’ to the end of the renilla luciferase gene and used to check the effect of miR-124 on renilla luciferase activity. The 3’UTR of MCP-1 and its coding regions were amplified from cDNA derived from RAW264.7 cells and cloned into psiCheck-2 (Life Technologies). The luciferase reporter plasmids pre-3031, pre-2116, pre-971 or pre-493 were constructed with PCR amplification using genomic DNA from mouse lungs tissue as template and subsequently cloned into pGL3-basic (Promega). The mutated 3’UTR of MCP-1 and miR-124 promoter constructs containing site-specific mutations at transcription factor binding sites were generated with overlap-extension PCR. Primers are listed in Table 6. All constructs were verified with sequencing.

**Table 6:** Sequence of primer dual luciferase plasmid construction

Primer	Forward (5’ to 3’)	Reverse (5’ to 3’)
MCP-1 3’ UTR	CGCTCGAGAGTATGAACTTTATT	TATAGCGGCCGCGGATCTTCAAA
MCP-1 3’ UTR MUT	TGTACTCATTCCGTCATTGT	ACATGAGTAAGGCAGTAACA
MCP-1 3’ UTR KNO	TGCTTTGTACTTCATTGTAATTA	ACGAAACATGAAGTAACATTAAT
Pre-3031	GGGGAGTCCAGGGAAGAAC	
Pre-2116	CGTGGGTGGTTTCCTTGACTTT	
Pre-1323	TGATAATCGCCCGGTGCC	
Pre-493	TCTCCGAACATCGAGGTTCTTT	
Anti-Uni-pre		GCTGTGAACACGCAGAGGG
SP-1 BS1 MUT	CGGAGTTGGTAAATTACCAGG	CCTGGTAATTTACCAACTCCG
SP-1 BS2 MUT	GCCCTGATATTAACTCTGCGT	ACGCAGAGTTAATATCAGGGC

### Luciferase reporter assay

RAW264.7 cells were co-transfected with 50 ng psiCheck-2 with insertion of the wild-type, mutant, or knockout 3’UTR of MCP-1, along with miR-124 mimics, inhibitors or controls (50nmol/L). After 24h, the cells were collected for use in the Dual-Luciferase Reporter Assay (Promega). For miR-124 promoter analysis, RAW264.7 cells were co-transfected with 100 ng full length, truncated or mutant promoter firefly luciferase reporter constructs and 10 ng Renilla luciferase vector (pRL-TK). Luciferase activities were determined and were expressed as relative luciferase activity by normalizing firefly luciferase activity to Renilla luciferase activity.

### Western blotting and ELISA

Whole-cell lysates were generated in 1 mL of ice-cold lysis buffer (1× Tris-buffered saline, 1.5%Triton X-100, 0.5% deoxycholic acid, 0.1% SDS, protease inhibitor cocktail, and 1mM PMSF). After centrifugation (12,000 g, 20min, 4°C), the supernatants were collected for western blotting. Protein concentration was determined with Bio-Rad Dc protein assay solution. Then protein samples were loaded onto SDS-polyacrylamide gel and transferred to nitrocellulose membranes. The membranes were blocked and then incubated with primary antibodies specific for ICAM-1, MCP-1, Sp1 and GAPDH at 4°C overnight and then with secondary antibodies labeled with horseradish peroxidase-conjugated goat anti-rabbit or goat anti-mouse IgG at room temperature. The signals were detected using enhanced chemiluminescence reagents (Thermo Scientific).

To measure the MCP-1 production in the transfected RAW264.7 cells, the cell culture medium was collected for ELISA. The antibody against MCP-1 was used as the primary antibody after blocking. After horseradish peroxidase (HRP)-conjugated secondary antibody incubation and washing, 3,3′,5,5′-Tetramethylbenzidine (TMB) (Solarbio Life Science, Beijing, China) substrate solution was used for enzyme reaction. After sufficient color developments add stop solution and read the optical density at 450 nm with ELX800 Bio-Tek.

### Myeloperoxidase (MPO) activity assay

To determine neutrophil infiltration, MPO activity of lung lysates was measured. Mouse lungs were homogenized in 0.5% hexadecyltrimethylammonium bromide dissolved in 10 mM potassium phosphate buffer (pH 7). After centrifugation at 20,000 g, aliquots of the supernatant were assessed for total protein concentration and MPO activity. An aliquot of each sample (supernatant) was loaded into each well of a 96-well plate, and o-dianisidinedihydrochloride with 0.0005% hydrogen peroxide in phosphate buffer was added to the samples. Absorbance was read at 460nm for 3min. MPO activity is expressed as the change in absorbance per minute per milligram of tissue.

### Histological assay

Formalin-fixed lung tissues were deparaffinized in xylene, rehydrated in graded alcohols, and stained with hematoxylin and eosin. The sections were evaluated for loss of normal lung architecture, erythrocyte exudation, and immune cell infiltration when treated with tail-vein LPS and miR-124 mimics. To further test the MCP-1 expression in lungs, immunohistochemistry experiments were conducted using a specific polyclonal anti-MCP-1 antibody. All sections were analyzed with a Nikon 80i microscope.

### Statistical analysis

All the experiments were performed in 3 sets unless otherwise indicated, values are means ± SEM. Statistical differences between groups were determined using AVONA or student’s *t*-test, depending on group size. P<0.05 was considered significant.

## SUPPLEMENTARY MATERIALS FIGURE


