# Supplementary material for: ICAM-1 regulates macrophage polarization by suppressing MCP-1 expression via miR-124 upregulation
Source: Oncotarget. 2017 Dec 5;8(67):111882–901. doi: 10.18632/oncotarget.22948 (PMC5762366; doi:10.18632/oncotarget.22948)
Supplement: Supplementary file 1 [file oncotarget-08-111882-s001.pdf]

ICAM-1 regulates macrophage polarization by suppressing MCP-1 expression via miR-124 upregulation

SUPPLEMENTARY MATERIALS

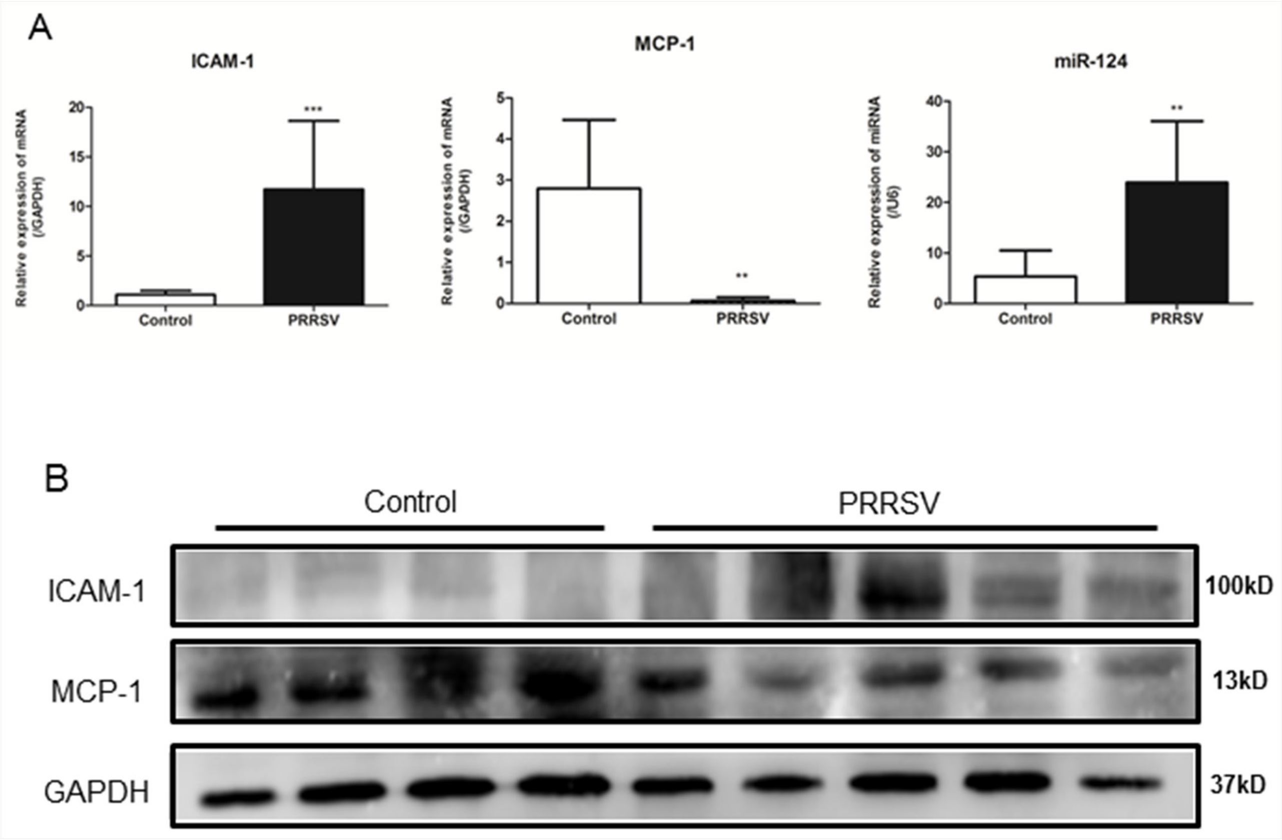

Supplementary Figure 1: Expression of ICAM-1, MCP-1 and miR-124 in PRRSV-infected swine lungs. **(A)** ICAM-1, MCP-1 and miR-124 mRNA expressions were determined by real-time qPCR. The data in graph were shown in mean ± SEM of 4 animals. **(B)** Protein expressions of ICAM-1 and MCP-1 were determined by Western blotting (n=4 for each groups ** p <0.01, *** p <0.001).
